# Supplementary material for: An Octopus-Derived Peptide with Antidiuretic Activity in Rats
Source: Mar Drugs. 2022 May 17;20(5):328. doi: 10.3390/md20050328 (PMC9144402; doi:10.3390/md20050328)
Supplement: Supplementary file 1 [file marinedrugs-20-00328-s001.zip › marinedrugs-1721832-supplementary.pdf]

Supplementary Material

TABLE OF CONTENTS

SUPPLEMENTARY FIGURES……………………………………………………………………..1

Figure S1. Optimal condition for weight of rats in the antidiuretic test. ...……………………………2

Figure S2. Optimal condition for breeding cage of rats in the antidiuretic test...……………………..3

Figure S3. Optimal condition for fasting time of rats in the antidiuretic test…….…………………..…..4

SUPPLEMENTARY FIGURES

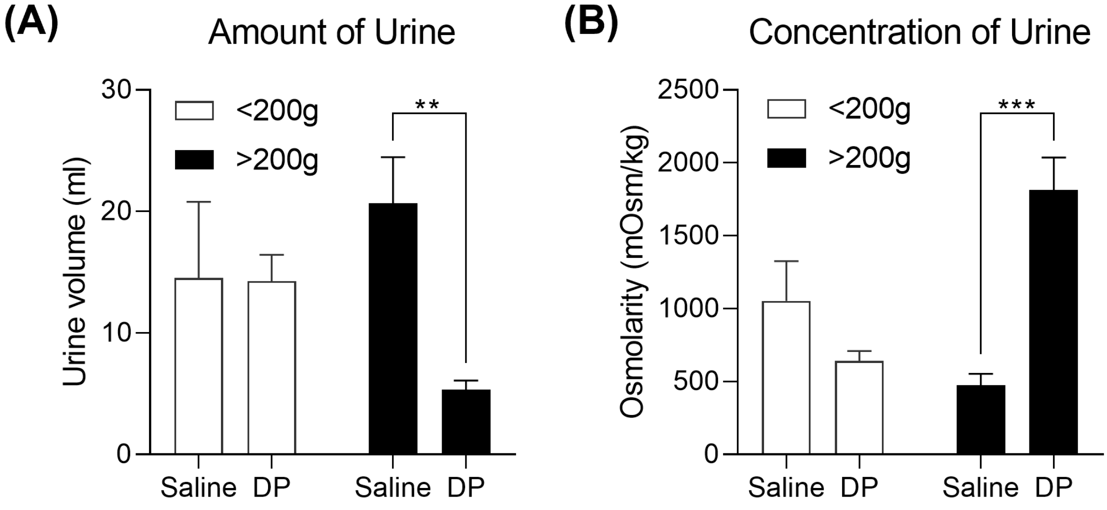

Figure S1. Optimal rat weight for the antidiuretic effect. (A) Male rats above 200 g produced less urine after a single microvenous injection of desmopressin (DP); however, male rats under 200 g exhibited no change in urine volume (unpaired t test **P = 0.0026, n=6 for each group). (B) Male rats above 200 g had lower osmolarity after a single microvenous injection of desmopressin (DP); however, male rats under 200 g had no change in osmolarity (unpaired t test ***P = 0.0002, n=6 for each group).

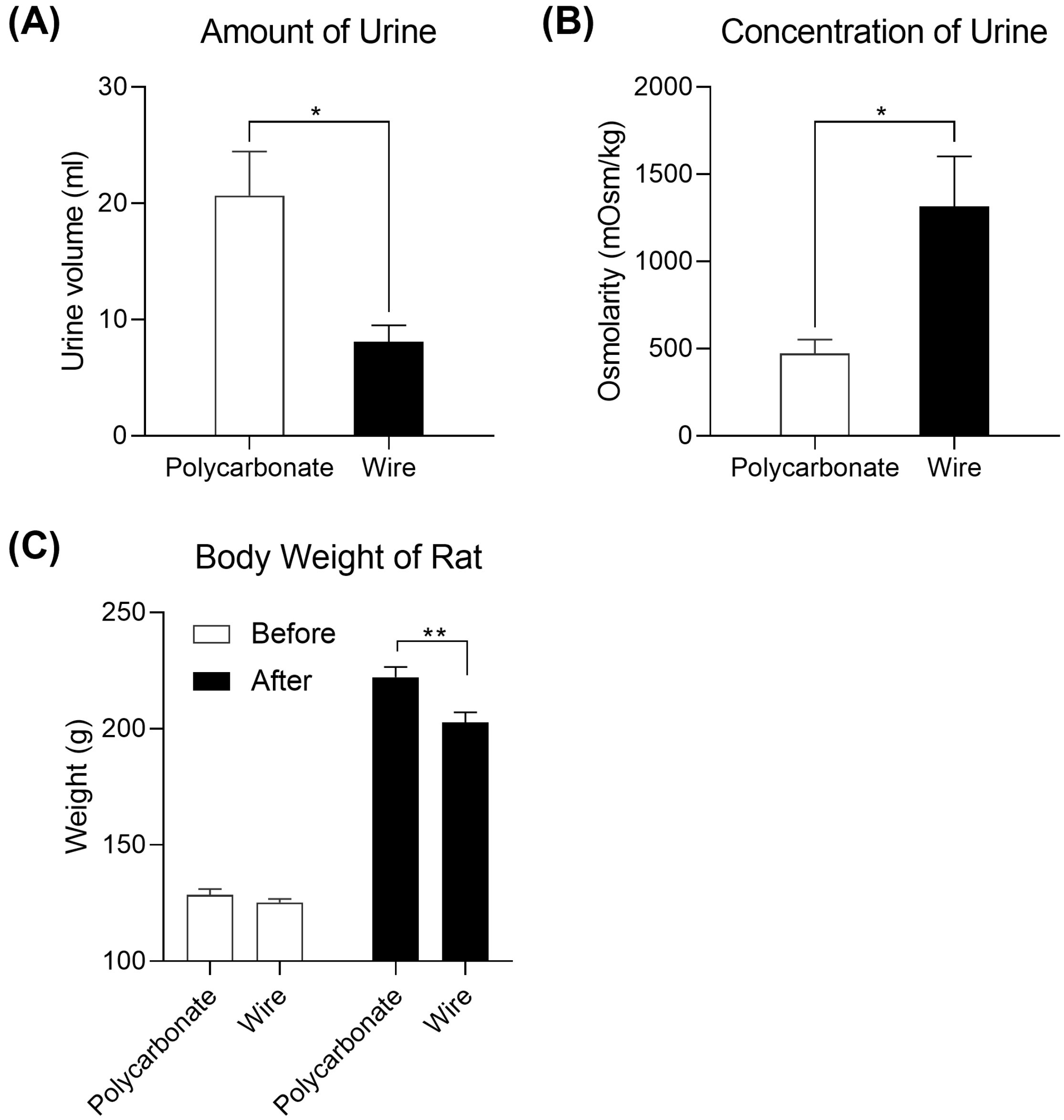

Figure S2. The optimal housing rat cage conditions for antidiuretic effects. (A,B) Male rats housed in polycarbonate-bottomed cages had different urine volumes (A, unpaired t test **P = 0.01, n=6 for each group) and osmotic pressures (B, unpaired t test *P = 0.02, n=6 for each group) than rats breeding in wire-bottomed cages. (C) Rats kept in wire-bottom cages weighed less than those kept in polycarbonate cages (unpaired t test **P = 0.0099, n=6 for each group).

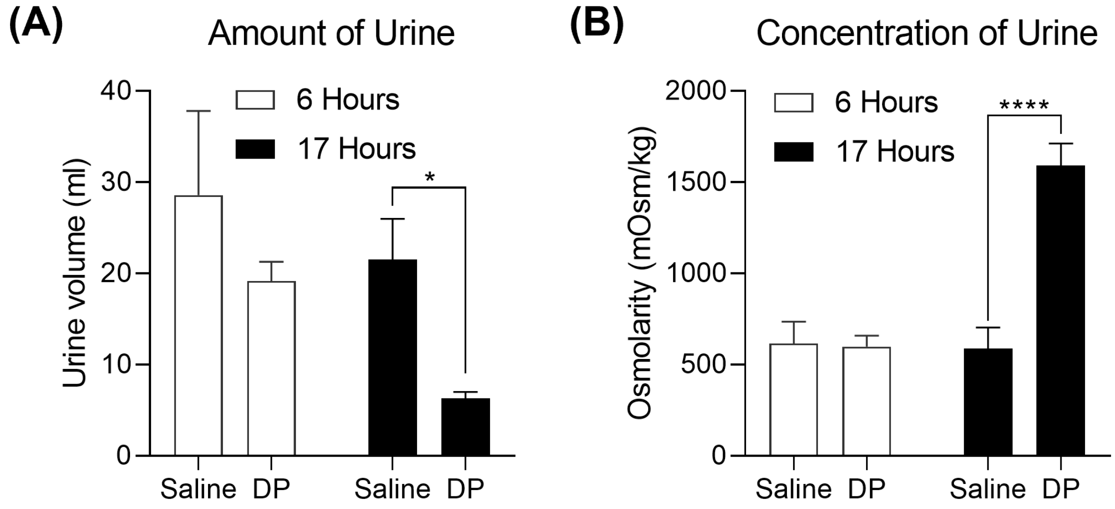

Figure S3. The optimal fasting time for the antidiuretic effect in rats. Time-dependent variation in dietary inhibition prior to microintravenous administration of desmopressin (DP). (A,B) The 17-hour fasting time showed DP-reduced urine volume (A, unpaired t test *P = 0.0112, n=6 for 6 hours and n=8 for 17 hours) and osmolarity (B, unpaired t test ****P < 0.0001, n=6 for 6 hours and n=8 for 17 hours)
